# The Child’s Mouth from the Standpoint of the Physician and Dentist

**Published:** 1912-10-15

**Authors:** J. E. Hunt

**Affiliations:** Kansas City, Mo.


### THE CHILD'S MOUTH FROM THE STANDPOINT OF THE PHYSICIAN AND DENTIST.

J. E. HUNT, M.D., KANSAS CITY, MO.

I feel quite sure that there is no other part of the child's anatomy which receives as little attention by both dentist and physician as the mouth.

Beginning with the infant—little or no instruction is given to either mother or nurse, regarding oral hygiene. And as a result the helpless babe soon develops a painful stomatitis which makes nursing well nigh impossible. The doctor is called and orders calomel and soda and goes his way rejoicing, while the little sufferer suffers on, only in a more marked degree on account of the irritation from the calomel.

Later on the gums begin to broaden and finally to swell as the little tooth is gradually pushing its way upward.

With the nervousness produced from this process the resistance of the child is lowered and any slight exposure or indiscretion in diet results in a gastro-intestinal upset which, according to many, can only be cured by lancing the gums.

Gum-lancing is popular with many and indeed is a remnant of the old days when little or nothing was known of children and their ills. But to-day, with our increased knowledge and also remembering that the tooth does not penetrate the gum, but the gum is absorbed away from it, this absorption being stimulated by the pressure of the up-coming tooth; the operation becomes unphysiological, yet with this physiological fact against the operation and the further fact of scar formation, which only retards further the process, I say, in spite of these facts, there are occasionally instances where the tooth is already above the gum line, but still covered and oedematous, a free incision, often relieves a distressing condition. But in the very rare instances where a free incision through an oedematous gum seems to be justifiable, the usual surgical neglect of this cut surface should not be tolerated. This incision needs to be kept as clean as a cut finger, but how often is it?

The teeth are now in and the possessor a babe no longer, but a child on a general diet, and with these new conditions what do we find?

Here in Kansas City, and after a most careful inspection, it was found that fully two-thirds of the children in the public schools had one or more decayed teeth and an equally constant gingivitis, many to the extent of gum erosion. Also the number of children with teeth missing from extraction was enormous.

Such conditions as these point to some very radical defects; first, in the home; second, in the kind or amount of instruction given by the family physician, and, third, with the dentist and his willingness to sacrifice a tooth rather than spend a little extra time over it and thus save it.

It is often a source of surprise to me to find many mothers of the wealthy classes quite ignoring the necessity for any sort of systematic oral hygiene for their children. And if this is so with such as these, how much more will it be, with the less fortunate and the generally ignorant? Many a child's digestion is ruined by a mouth full of decayed teeth. And, too, many a child's mouth is permanently deformed by a premature extraction of decayed teeth.

I realize the difficulties underlying a campaign for personal oral cleanliness, especially when many mothers will appreciate the value of clean ears and ignore the much more important mouth and teeth. But the greater the difficulty the greater the necessity, and only constant reiteration on the part of teachers and physicians will overcome this sad neglect.

As to the part our dental brethren play in this matter—with the knowledge at hand, I am forced to the conclusion that the vast majority of dentists either have not the ability or the inclination to do what they should for these little patients.

True, it takes both skill and tact and time, but when the health of a child or a permanent mouth deformity, from slipshod work, is at stake a greater willingness on the part of the dental profession should be exhibited.

A fault as universal as this must have a definite source of origin, and I take it that this place is the dental school.

A child enters a dental infirmary, nobody wants it and the instructor assigns the little patient to the poorest man in the class, with the observation that any kind of work is good enough for a child, and as a result the student mind is permanently impressed with the same idea and he goes forth into the dental world with this same fixed delusion, feeling that a child's mouth is beneath him. So that today mothers are constantly asking where there is a dentist who will do good work on their children's teeth with the added observation that their own puts them off whenever the subject is brought up. A sad confession indeed.

The remedy—give the student actual practical instruction in both prophylactic and operative dentistry as applied to the young.

It will be noticed that the above remedy is not qualified by the word "more." Indeed, judging from the character of the local infirmary work on children, I am quite convinced that the student receives no such instruction, so why qualify by asking for more instruction?—*Western Dental Journal.*

